# Intrabiliary growth type of metastasis from colon cancer, 12 years after curative colectomy: a case report

**DOI:** 10.1186/s12893-018-0466-4

**Published:** 2019-01-18

**Authors:** Shin Sasaki, Yoriko Nomura, Shogo Fukutomi, Nobuhisa Shirahama, Hironori Kusano, Jun Akiba, Hisamune Sakai, Toru Hisaka, Osamu Nakashima, Hirohisa Yano, Yoshito Akagi, Hiroyuki Tanaka, Koji Okuda

**Affiliations:** 1 Department of Surgery, Kurume University School of Medicine, 67 Asahi-machi, Kurume, 8300011 Japan; 2 Department of Pathology, Kurume University School of Medicine, 67 Asahi-machi, Kurume, 8300011 Japan; 3 Department of Diagnostic Pathology, Kurume University Hospital, 67 Asahi-machi, Kurume, 8300011 Japan; 4 Clinical Laboratory, Kurume University Hospital, 67 Asahi-machi, Kurume, 8300011 Japan; 5 Division of Hepatobiliary and Pancreatic Surgery, Department of Surgery, Kurume University School of Medicine, 67 Asahi-machi, Kurume, 8300011 Japan

**Keywords:** Intrabiliary growth type of metastasis, IGM, Metastasis, Colorectal cancer, Hepatectomy, Liver

## Abstract

**Background:**

Liver is a common location of colorectal metastasis, but intrabiliary growth of liver metastasis is not well recognized. Furthermore, intrabiliary metastasis that discovered over 10 years after excision has rarely been described.

**Case presentation:**

An 80-year-old man was admitted due to the presence of a liver mass in segment 5 (S5) concomitant with elevated carcinoembryonic antigen (CEA), and carbohydrate antigen (CA) 19–9. He underwent right hemicolectomy for colon cancer 12 years prior. Enhanced computed tomography (CT) showed dilated bile ducts with periductal enhancement in S5; hence, cholangiocarcinoma was suspected. Upon anterior segmentectomy, we observed that the cut surface of the specimen exhibited a yellowish-white tumor within the bile ducts. Histologically, the tumor formed within the papillary process, extended along the lumen, and replaced the normal bile duct epithelium. Immunohistochemical studies showed that the liver tumor and primary colon cancer were negative for cytokeratin (CK) 7 and positive for CK20 and Caudal-type homeobox transcription factor 2 (CDX-2). In addition, both tumors showed a same KRAS mutation. We diagnosed the liver tumor as liver metastasis recurrence from colon cancer.

**Conclusion:**

Intrabiliary growth type of metastasis (IGM) is difficult to distinguish from cholangiocarcinoma, and sometimes develops long after surgery; thus, careful examination of a patient’s history is needed in such cases.

## Background

Liver metastasis from colorectal cancer that develops more than 5 years after curative colectomy is extremely rare (0.1%) in Japan [1]. Furthermore, liver metastasis from colorectal cancer usually forms nodular mass, whereas intrabiliary papillary growth in the bile duct is unusual and not well recognized. The intrabiliary growth type of metastasis (IGM) resembles the intraductal growth type of intrahepatic cholangiocarcinoma, thereby resulting in liver metastasis being misdiagnosed as cholangiocarcinoma [2]. Immunohistochemical study is useful for distinguishing between liver metastases and cholangiocarcinoma [3–7]. Here in, we report a case of IGM that appeared 12 years after the resection of primary colorectal cancer.

## Case presentation

### Patient

An 80-year-old man was admitted to our hospital due to the presence of a liver mass in segment 5 (S5). He had undergone right hemicolectomy for colon cancer 12 years prior; his condition was pathologically diagnosed as well-to-moderately differentiated adenocarcinoma with lymph node metastasis and venous invasion. A total of 22 lymph nodes were resected during the initial surgery. Among these, six lymph nodes were positive for metastasis. The tumor was classified as stage IIIb. After right hemicolectomy, he was administered adjuvant chemotherapy of 5-fluorouracil for 1 month, but the treatment was discontinued because of adverse drug events. During the 12 years of follow up, there was no local recurrence. After the 5-years follow-up period, this patient was followed-up by his primary care physician. Tumor markers were examined occasionally by his primary care physician, and computed tomography (CT) was performed because of the increase in the tumor marker levels. The liver mass was detected on CT. Then, the patient was referred to our hospital. Upon admission, his carcinoembryonic antigen (CEA) and carbohydrate antigen (CA) 19–9 levels were elevated to 21.4 ng/ml and 174.5 U/ml, respectively. However, α-fetoprotein (AFP) and protein induced by vitamin K absence-2 (PIVKA-2) levels were normal. Hepatitis B surface antigen and hepatitis C antibody titers were both negative.

### Dynamic computed tomography (CT)

**Fig. 1:**
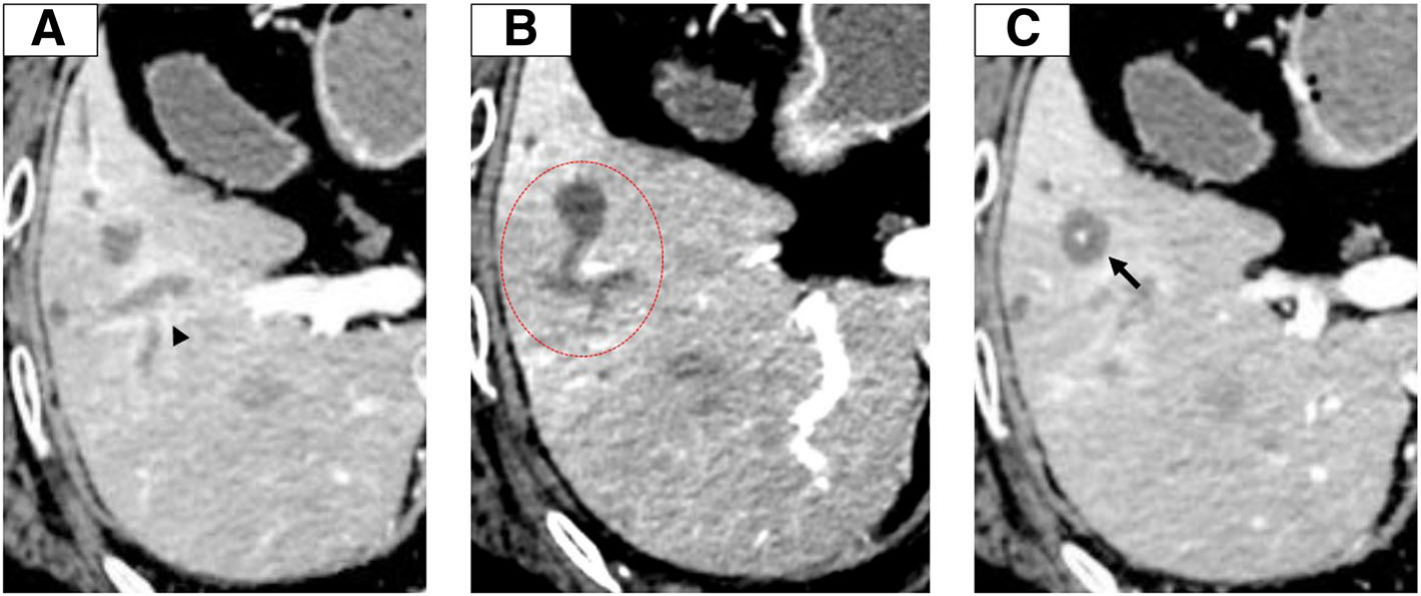
Continuous enhanced CT image from the cranial to caudal side. **a**: Periductal enhancement is observed along the dilated bile ducts (arrowhead). **b**, **c**: The peripheral branch of the continuous expanded bile duct exhibited a mass-like (**b**, red circle) region that contained a spotty high-density area that was observed even on plane-phase CT (**c**, arrow)

On enhanced CT, periductal enhancement was observed along the dilated bile ducts in the portal and venous phase (Fig. 1 a, arrowhead); however, the accompanied mass, which causes peripheral biliary duct dilation, was not detected. The peripheral branch of the continuous expanded bile duct (Fig. 1 b, red circle) exhibited a mass-like appearance (S5) that contained a spotty high-density area that was observed even when using plane phase CT (Fig. 1c, arrow).

### Magnetic resonance imaging (MRI)

**Fig. 2:**
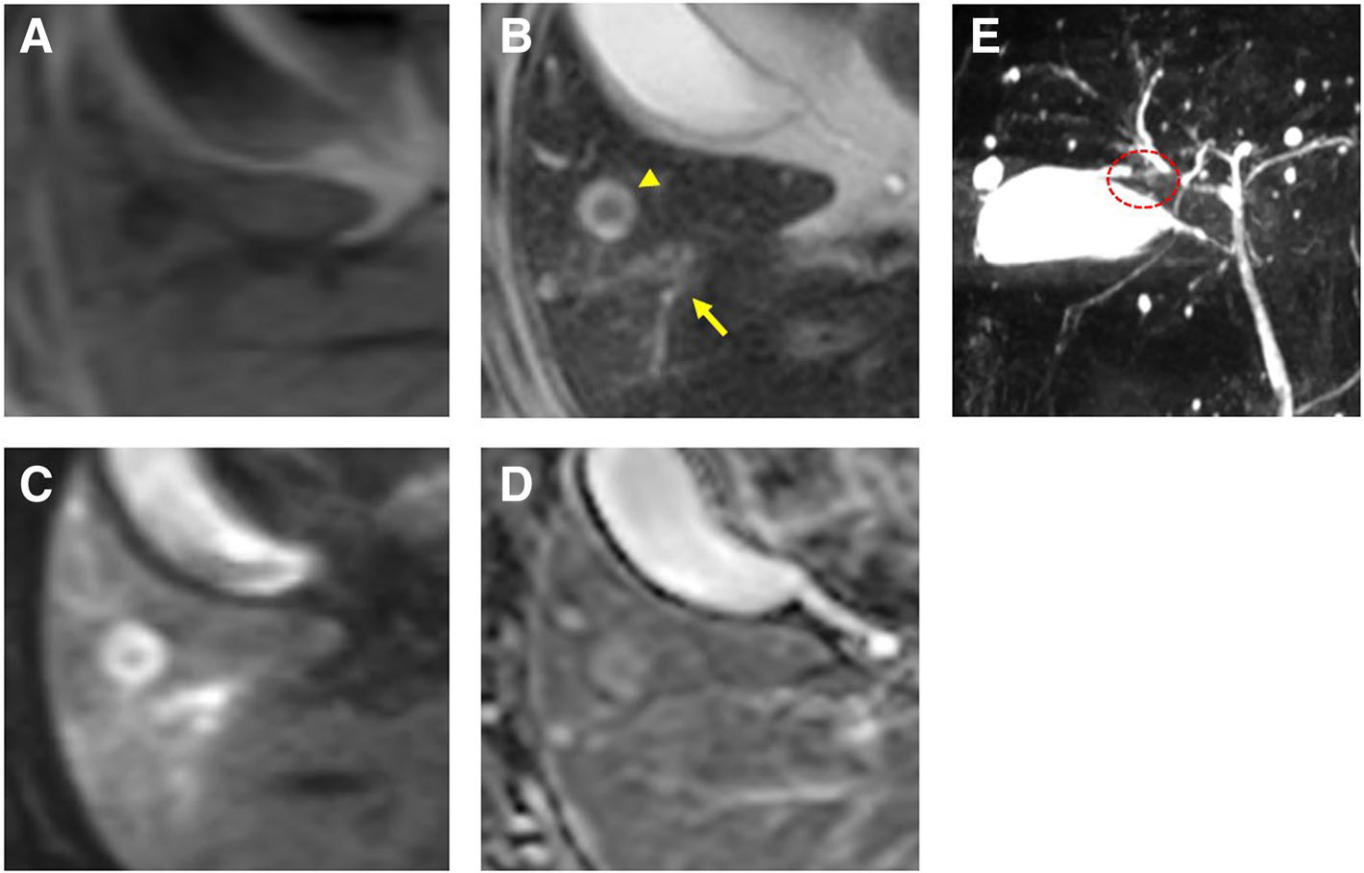
**a**-**e**:**a**: MRI T1WI: There was hypo-intensity around the dilated bile duct. **b**: MRI T2WI: Periductal high-intensity was observed around the dilated bile ducts (arrow) and the high-intensity area included a low-intensity area in S5 (arrowhead). **c**: MRI DWI: The dilated bile ducts showed periductal high intensity. **d**: MRI ADC map: Slight high-intensity was observed around the bile ducts. **e**: MRCP showed stenosis of the anterior-inferior branch of the bile duct (red circle)

There was hypo-intensity on T1 weighted image (Fig. 2a) around the dilated bile duct. On T2 weighted imaging, modestly high intensity was observed along the dilated bile ducts (Fig. 2b, arrow), with a high-intensity area that included low-intensity in the S5 (Fig. 2b, arrow head) being observed. The dilated bile ducts showed a high intensity on diffusion weighted images (DWI) (Fig. 2c). An apparent diffusion coefficient (ADC) map displayed slightly high-intensity visually (Fig. 2d). Magnetic resonance cholangiopancreatography (MRCP) showed stenosis of the anterior-inferior branch of the bile ducts (Fig. 2e, red circle) and the peripheral bile ducts were dilated.

### Positron emission tomography (PET)

**Fig. 3:**
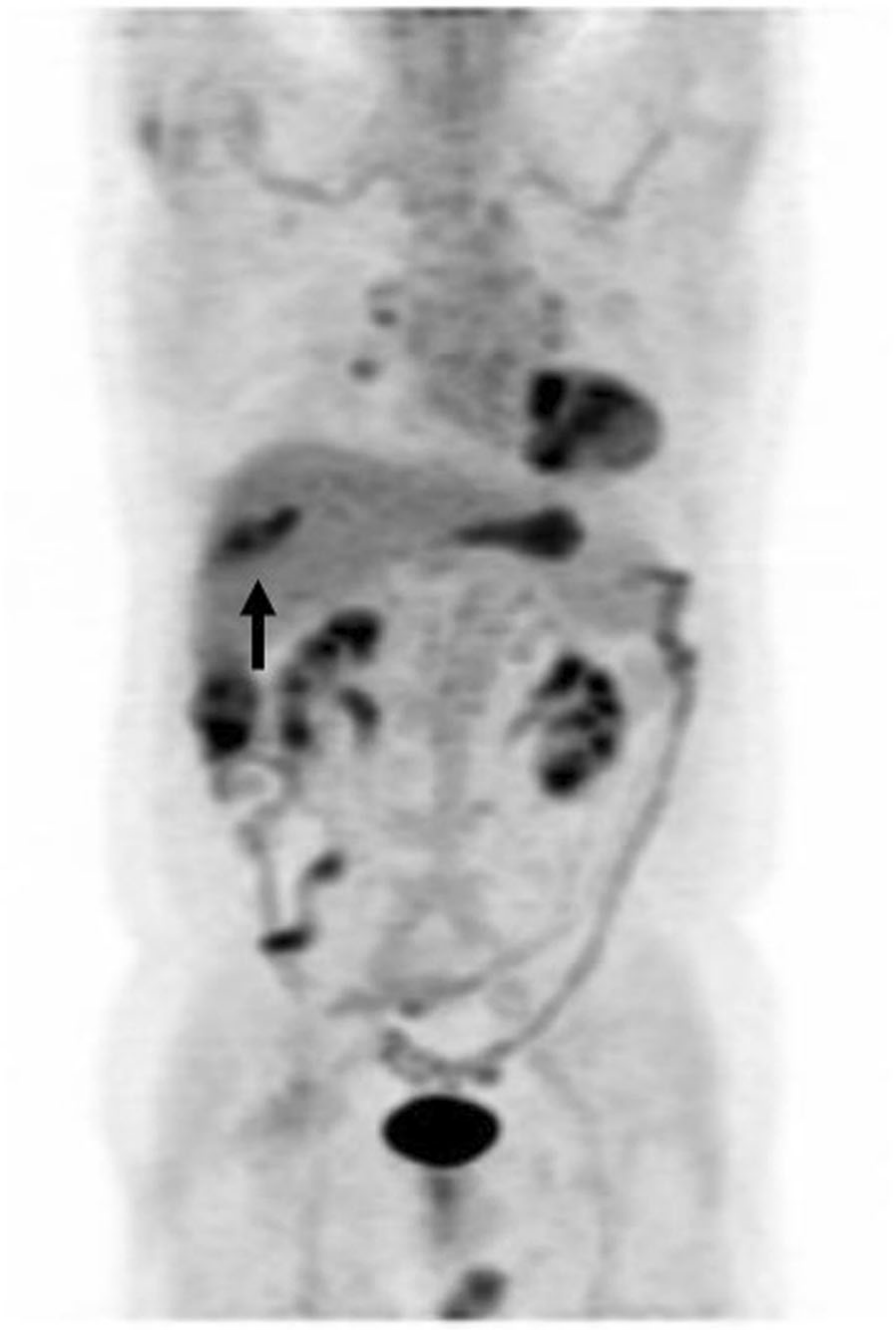
PET imaging revealed abnormal uptake in S5 that was consistent with the site of bile duct dilatation, as observed from CT/MRI findings (arrow)

Fludeoxyglucose F18 (_18_F-FDG) PET imaging revealed abnormal uptake in the liver that was consistent with the site of bile duct dilatation, as observed in CT/MRI findings (Fig. 3).

### Contrast enhanced ultrasonography (CEUS)

**Fig. 4:**
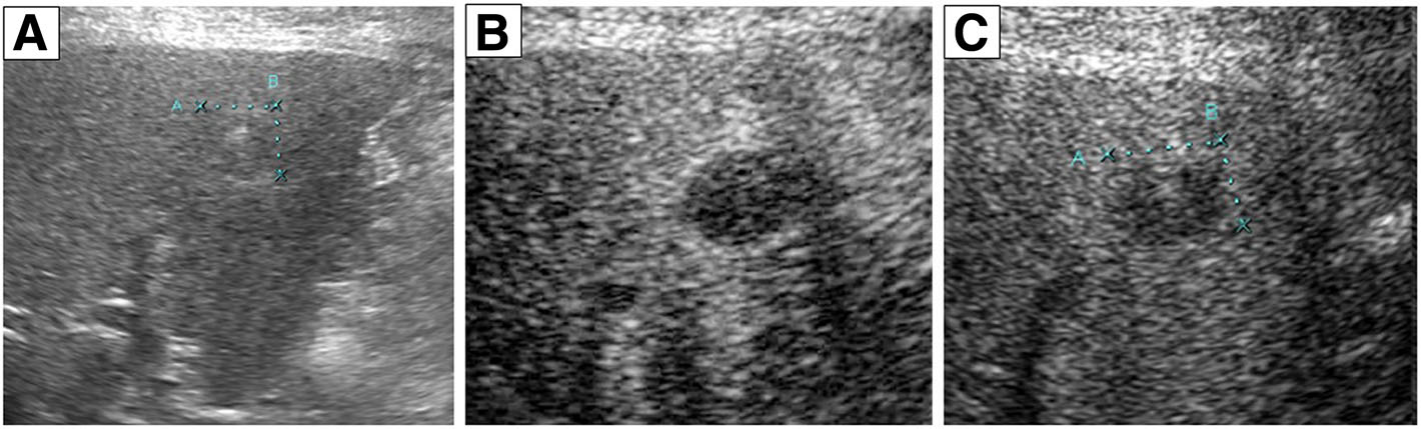
**a**: Ultrasonography showed intrahepatic bile duct dilatation and a low echoic lesion in S5. **b**,**c**: CEUS in the Vascular phase (**b**) and Kupffer phase (**c**) showed no enhancement

Ultrasonography (US) showed dilated bile ducts and a low echoic lesion 15 mm in diameter with no posterior echo enhancement in S5 (Fig. 4a). On CEUS, the low echoic lesion showed no enhancement in the vascular phase and remained low, even in the Kupffer phase (Fig. 4b, c).

### Endoscopic retrograde cholangiography (ERC)

**Fig. 5:**
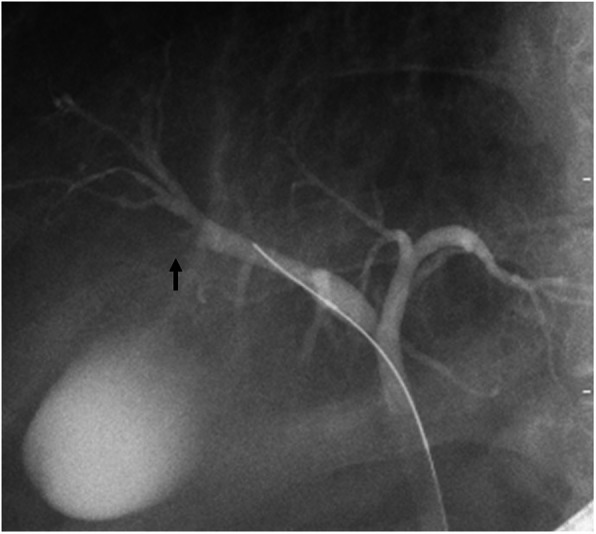
Endoscopic retrograde cholangiography (ERC) revealed interruption of the anterior-inferior branch of the bile duct (arrow)

ERC confirmed interruption of the anterior-inferior branch of the bile duct (Fig. 5). We performed cytologic examination of the bile juice, but it was negative for malignant cells. Furthermore, intraductal biopsy was performed. The tissue obtained from anterior branch of the bile duct was adequate for histological diagnosis; however, the biopsy revealed no malignancy.

### Hepatectomy

We did not performed percutaneous biopsy because of the risk of dissenmination of tumor cells intraperitoneally. Preoperatively, we clinically diagnosed an intraductal growth type of intrahepatic cholangiocarcinoma. Therefore, after informed consent was obtained, anterior segmentectomy was performed.

### Macroscopic findings

**Fig. 6:**
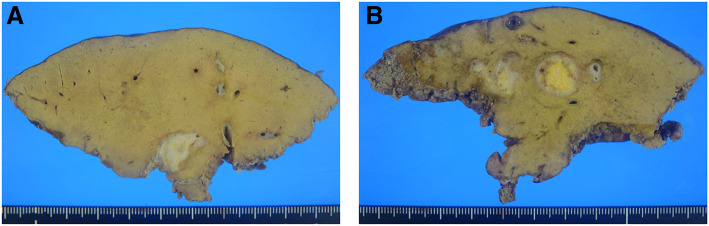
The bile duct wall was continuously thickened (**a**), and a part of the bile duct formed a yellowish-white solid nodule (**b**)

In the resected specimen, the anterior segment bile duct wall was continuously thickened, and a part of the bile duct formed a yellowish-white solid nodule that was 19 × 15 mm in diameter (Fig. 6a, b).

### Histological findings

**Fig. 7:**
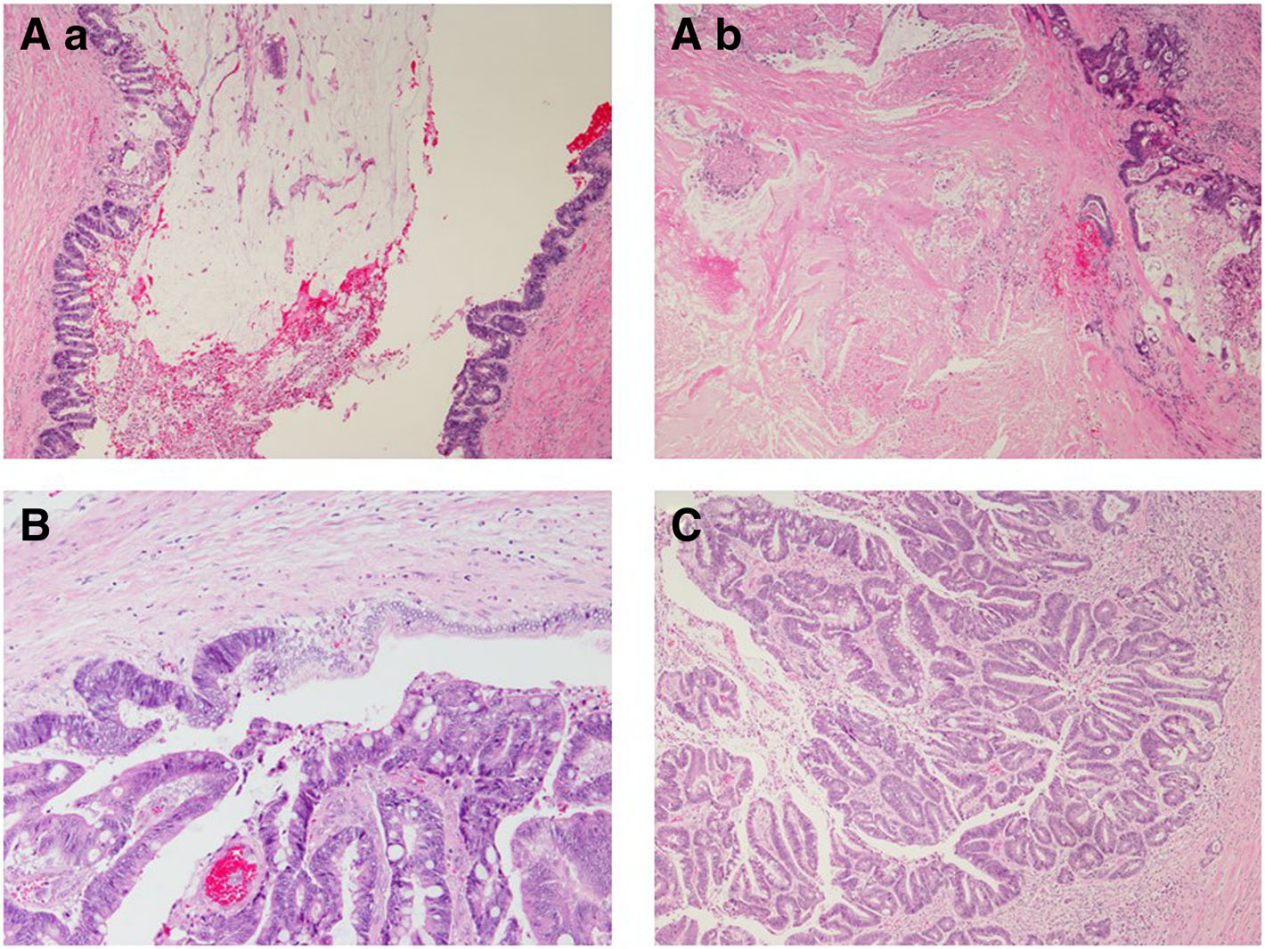
**a**: The bile duct was filled with a large amount of mucin (a) and the inside of the tumor-like part was full of necrotic tissue (b). **b**: The tumor cells along the lumen replaced the normal epithelium of the bile duct. **c**: The tumor cells formed papillae with fibrovascular cores inside the dilated bile duct, comprising columnar mucin-producing cells

Histological examination revealed that the bile duct was filled with a large amount of mucin. The inside of the tumor-like part was full of necrotic tissue (Fig. 7a). The tumor cells extended along the basement membrane and replaced the normal epithelium of the bile duct (Fig. 7b). Meanwhile, the tumor cells formed papillae with fibrovascular cores inside the dilated bile duct, which were comprised of columnar mucin-producing cells (Fig. 7c). There was no tumor invasion into the liver parenchyma. The liver tumor resembled colon cancer that was resected 12 years prior. Even with primary colon cancer, tumor cells formed papillae with mucinous production as well as bile duct lesions. Therefore, immunohistochemical studies of cytokeratin (CK) 7, CK20, and Caudal-type homeobox transcription factor 2 (CDX2) were performed to distinguish between cholangiocarcinoma and metastatic liver cancer.

**Fig. 8:**
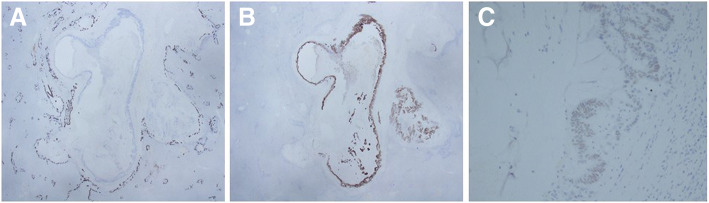
Immunohistochemical studies were negative for CK7 (**a**), positive for CK20 (**b**), and positive for CDX2 (**c**)

**Fig. 9:**
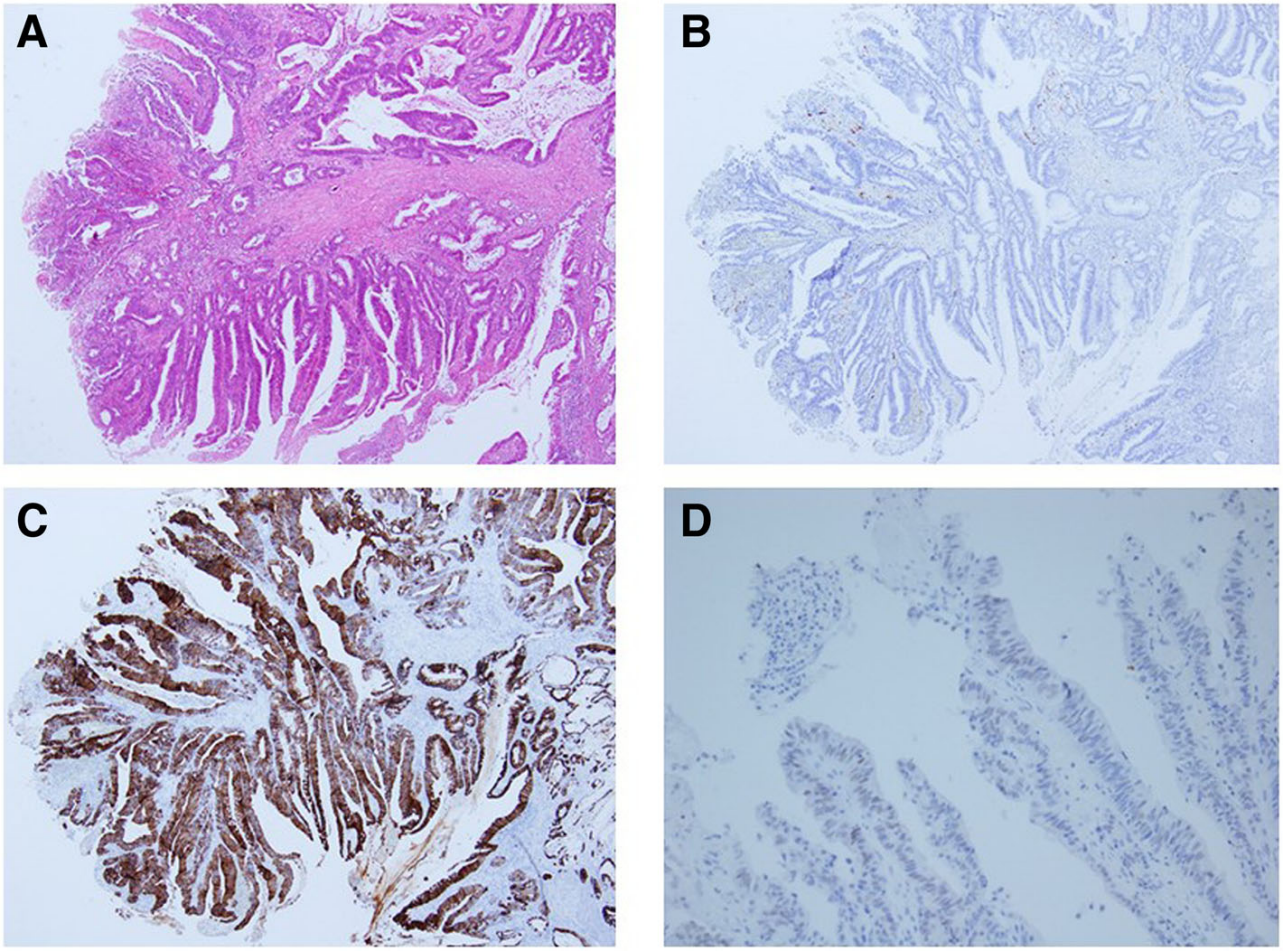
**a**: The primary colon cancer was confirmed as well-differentiated to moderately differentiated adenocarcinoma. **b**-**d**: Immunohistochemical staining in primary colon cancer was negative for CK7 (**b**), positive for CK20 (**c**), and positive for CDX2 (**d**)

Immunohistochemically, the tumor cells of the liver were positive for CK 20 and CDX-2, but negative for CK7 (Fig. 8a-c). Meanwhile, the primary colon cancer was also positive for CK20 and CDX-2, but negative for CK7 (Fig. 9a-d). Furthermore both tumors exhibited the same V-Ki-ras2 kirsten rat sarcoma viral oncogene homolog (KRAS) mutation in G12D. As a result, the liver tumors were diagnosed as intraductal papillary growth type that formed due to liver metastasis from colorectal cancer.

### After surgery

The postsurgical course was favorable, and the patient was discharged from the hospital 19 days after surgery. At 2 years after surgery, there were no signs of recurrence.

## Discussion and conclusions

Morphologically, liver metastasis from colorectal cancer often presents as an irregular tumor mass and sometimes infiltrates bile ducts, whereas intraductal papillary growth is usually confined to the bile duct and is not well recognized. IGM has been described in a few reports [2–4, 8–21]. However, there are few reports of recurrence after more than 10 years since primary resection. The period between the initial surgery and recurrence in our case is among the longest that has been reported to date [2, 3, 8–21].

IGM is more common with colon cancer than with other types of cancer [2]. The incidence of intrabiliary growth due to metastatic colon cancer ranges from 3.6 to 10.6% among all liver metastases from colon cancer. Meanwhile, metastasis from other types of cancer ranges from 0.7 to 1.9% [2]. IGM can be indistinguishable from the intraductal growth type of intrahepatic cholangiocarcinoma, which renders it sometimes difficult to make a correct diagnosis [3, 4]. Immunohistochemical staining for CK7, CK20, and CDX-2 are useful for identifying liver metastasis from cholangiocarcinoma [5, 6]. In colorectal carcinoma metastasis, the CK7-/CK20+ expression pattern had a 93% predictive positive value. Meanwhile, the CK7-/CK20+ profile has only a 4% predictive positive value for cholangiocarcinoma [5]. Moreover, CDX-2 is a highly specific and sensitive marker for colorectal origin, being expressed in 97% of colorectal cancers [6]. However, the positive predictive value of CDX-2 for cholangiocarcinoma ranged from 9 to 25% [7, 22]. In our case, we could not establish a diagnosis only based on radiological examination and hematoxylin-eosin staining. Upon immunohistochemical studies, the liver tumor was found to exhibit an expression pattern of CK7-/CK20+/CDX-2+. Moreover, there was a KRAS mutation in G12D. This pattern of expression was observed in the tumor cells that were resected from colon cancer 12 years prior. Therefore, we diagnosed the patient in our case with liver metastasis from colon cancer.

Previous studies have reported that intrabiliary growth type of liver metastasis from colon carcinoma, as summarized in Table 1 [2, 3, 8–21]. Most cases recurred within 5 years after primary curative surgery; however, some cases also recurred after more than 5 years. Thus, our case represents the longest interval from colon cancer resection to liver metastasis.

**Table 1:** Literature review of case reports or series involving intrabiliary growth of liver metastasis

Author (year)	Number of patients	Sex	Age (years)	^a^Interval	Histology of primary colon cancer	Prognosis
Kon et al. (2016) [20]	1	Male	62	72 months	Well	No evidence of recurrence 3 years after resection
Dong et al. (2016) [21]	1	Male	71	48 months	NA	N/A
Kawakatsu et al. (2015) [8]	1	Male	73	108 months	Well to mode	Intrapancreatic bile duct metastasis 12 years after hepatectomy
Coppola et al. (2014) [9]	1	Female	61	43 months	Mode	No evidence of recurrence 6 months after resection
Nakamura et al. (2013) [10]	1	Male	67	16 months	NA	No evidence of recurrence 14 months after resection
Ghittoni et al. (2010) [11]	1	Male	69	30 months	Well	No evidence of recurrence 12 months after resection
Nanashima et al. (2011) [3]	1	Male	65	15 months	Mode	NA
Hiramatsu et al. (2007) [12]	1	Male	77	36 months	Mode	Intrapancreatic bile duct metastasis 2 years later after hepatectomy
Takamatsu et al. (2004) [13]	1	Male	62	36 months	Mode	No evidence of recurrence 49 days after resection
Uehara et al. (2004) [14]	1	Male	72	14 months	Well	No evidence of recurrence 18 months after resection
Sano et al. (2000) [15]	1	Male	48	48 months	Mode	Intrapancreatic bile duct metastasis 3 years later after hepatectomy
Estrella et al. (2013) [2]	42	M/F: 21/21	54.9 (28–78)	28 months (0–139)	Well/mode/poor: 2/36/5	5 year OS: 33%
Sugiura et al. (2006) [16]	6	M/F: 5/1	56.3 (26–77)	35.8 months (0–109)	Well / mode: 4/2	Median survival after final hepatectomy were 35 months (8–93)
Kubo et al. (2002) [17]	8	M/F: 4/4	54.8 (43–64)	37.4 months (0–83)	Well: 8	NA
Okano et al. (1999) [18]	18	M/F: 12/6	65 (47–75)	30 months (12–84)	well/mode: 12/6	5-year OS: 80%
Riopel et al. (1997) [19]	8	M/F: 4/4	NA	45 months (0–113)	NA	NA

*M* male, *F* female, *well* well differentiated adenocarcinoma, *mode* moderately differentiated adenocarcinoma, *poor* poorly differentiated carcinoma, *OS* overall survival, *NA* not available

^a^ Interval: The period from resection of primary colon cancer or surgery of metastasis to recurrence

As shown in Table 1, histological analysis of the primary cancer types revealed that most were well or moderately differentiated adenocarcinoma. Kubo et al. reported that macroscopic intrabiliary extension tumors showed less aggressive features, with most primary tumors revealing well-differentiated adenocarcinoma and a few exhibiting venous invasion. Moreover, they described that the interval between colectomy and hepatectomy of the macroscopic intrabiliary type is longer than that type in which duct invasion is not observed [17]. IGM exhibits low invasiveness and is considered to have a longer interval of recurrence [18]. The prognosis that has been reported in these studies has varied, with Okano et al. having reported that patients with IGM exhibiting a good survival rate compared to patients with no bile duct invasion. Meanwhile, Estrella et al. observed that there was no significant difference between these two types [2, 18].

**Fig. 10:**
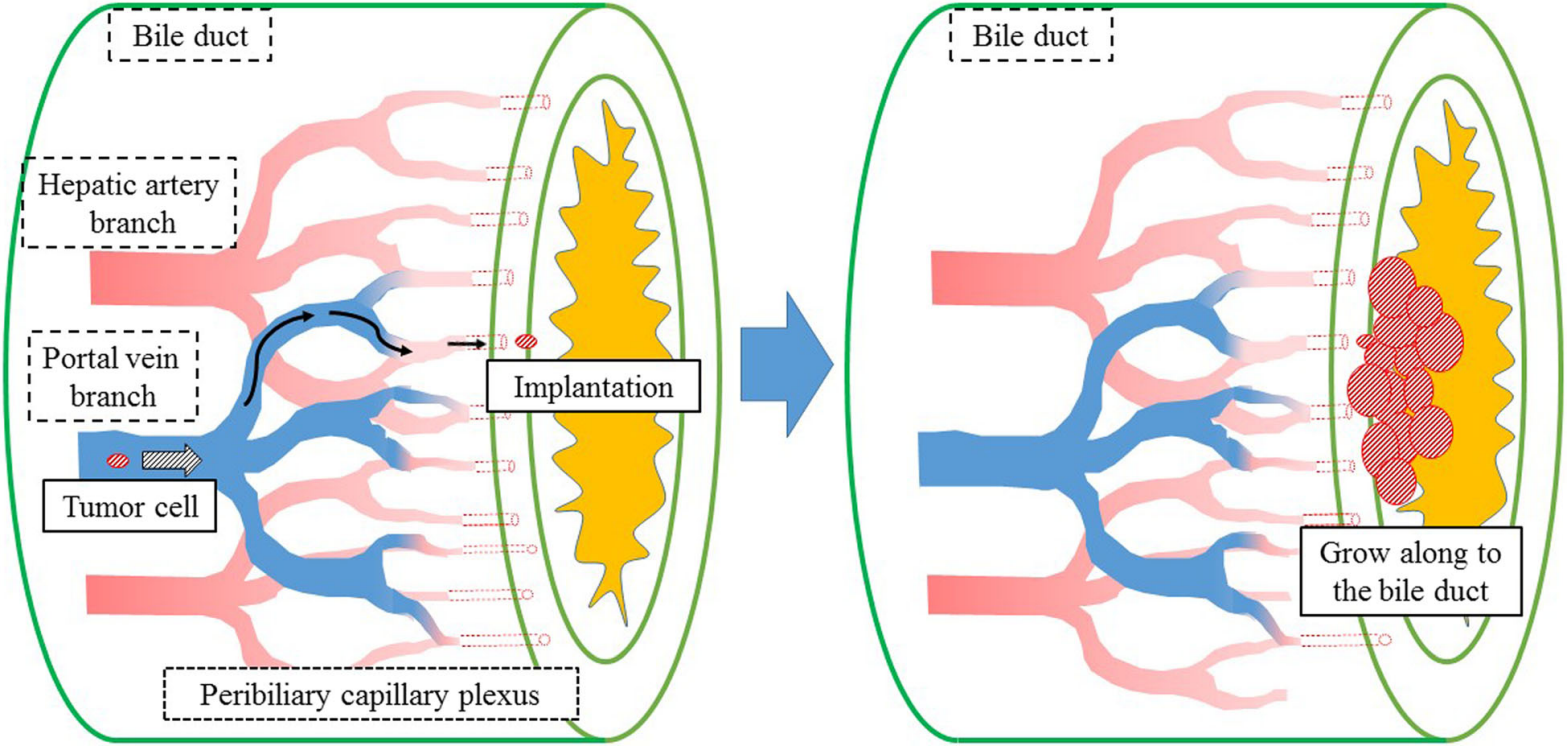
Mechanism of intrahepatic bile ducts metastasis from colorectal cancer. Cancer cells may spread from the primary colorectal cancer via the portal circulation and implant in the bile duct by passing through the peribiliary capillary plexus. (Source: Author’s own fugure)

The mechanism of IGM from colorectal cancer is unknown. Previous reports have described that liver metastasis from colon cancer has a higher affinity for bile ducts than for the parenchyma [11, 17]. We suspected that cancer cells could spread from primary colorectal cancer via portal circulation, before ultimately undergoing implantation to the peribiliary site by passing through the capillary plexus. Furthermore, the carcinoma grew along the bile ducts, which logically follows from the affinity of colon cancer for bile ducts (Fig. 10). Histological examination of the primary colon cancer revealed well-differentiated to moderately differentiated adenocarcinoma with lymphatic and venous invasion. (depth ss, ly2, v1, aw(−), ow(−), n(+) [#201 5/15, #202 1/4, #211 0/2, #213 0/1]; Japanese Classification of Colorectal Carcinoma). The metastatic lesion revealed micro-venous invasion (s0, vp1, vv0, va0, b3; General Rules for the Clinical and Pathological Study of Primary Liver Cancer). The primary lesion and metastatic lesion showed venous invasion. This result may help to explain the mechanism of IGM from colon cancer via the portal vein. The cancer observed in this case similarly exhibited low invasiveness and recurred long after the primary excision was performed. Furthermore, other reports have indicated that such cancers can be distributed via the hepatic artery and bile ducts [8, 15]. However, the molecular mechanism of intrahepatic bile duct metastasis is unclear and so further clinical findings are needed.

This patient was not administered adjuvant chemotherapy after liver resection. After the initial surgery, the patient was administered adjuvant chemotherapy, but the treatment had to be discontinued because of adverse drug events. Because of the adverse events before, the patients did not want adjuvant chemotherapy after hepatectomy. In some reports revealed many risk factor for recurrence, such as number of tumors, preoperative CEA level, tumor size, lymph metastasis and surgical margin [23]. Furthermore, many scoring system have been used for colorectal liver metastasis, but the precision of these system is unclear [23–25]. It remains controversial whether adjuvant chemotherapy should be administered after liver resection even the consensus has not been reached regarding the necessity of adjuvant chemotherapy after liver resection.

In conclusion, we present a rare case of metastatic carcinoma of the liver from colon cancer that exhibited intrabiliary papillary growth and occurred 12 years after curative colectomy. IGM is difficult to distinguish from cholangiocarcinoma, but immunohistochemical staining for CK7, CK20, and CDX-2 is helpful in achieving a correct diagnosis. Furthermore, IGM from colon cancer occasionally occurs long after the primary excision is performed; thus, careful examination of a patient’s history is needed in such cases.

## Abbreviations

_18_F-FDG: Fludeoxyglucose F18
ADC: Apparent diffusion coefficient
AFP: α-fetoprotein
CA 19-9: Carbohydrate antigen
CDX2: Caudal-type homeobox transcription factor 2
CEA: Carcinoembryonic antigen
CEUS: Contrast enhanced ultrasonography
CK: Cytokeratin
CT: Computed tomography
DWI: Diffusion weighted images
ERC: Endoscopic retrograde cholangiography
IGM: Intrabiliary growth type of metastasis
KRAS: V-Ki-ras2 kirsten rat sarcoma viral oncogene homolog
MRCP: Magnetic resonance cholangiopancreatography
MRI: Magnetic resonance imaging
PET: Positron emission tomography
PIVKA-2: Vitamin K absence-2
S5: Segment 5
